# Supplementary material for: Response of Lactiplantibacillus plantarum NMGL2 to Combinational Cold and Acid Stresses during Storage of Fermented Milk as Analyzed by Data-Independent Acquisition Proteomics
Source: Foods. 2021 Jun 30;10(7):1514. doi: 10.3390/foods10071514 (PMC8305577; doi:10.3390/foods10071514)
Supplement: Supplementary file 1 [file foods-10-01514-s001.zip › foods-1246954-supplementary.pdf]

**Table S1.** Growth of 31 strains of lactic acid bacteria isolated from traditional fermented products in MRS broth at 37°C for 18 h as shown with viable bacterial counts ($n$ = 3, $x$ ± SD).

| **Strain** | **Viable bacterial count(log cfu/ml)** | **Strain** | **Viable bacterial count(log cfu/ml)** | **Strain** | **Viable bacterial count(log cfu/ml)** |
|---|---|---|---|---|---|
| ***Lpb. pentosus* PC11** | 10.35±0.27 | ***Lpb. plantarum* P6** | 9.65±0.15 | ***Lpb. pentosus* PC1** | 6.93±0.10 |
| ***Lpb. plantarum* NMGL2** | 10.19±0.15 | ***Lcb. casei* JS2** | 9.61±0.09 | ***Lpb. plantarum* NMGL4** | 6.86±0.23 |
| ***Lpb. plantarum* P4** | 10.18±0.04 | ***Lcb. casei* JS1** | 9.57±0.20 | ***Lab. brevis* JS5** | 6.37±0.39 |
| ***Lpb. plantarum* NMGL1** | 10.14±0.15 | ***Lab. brevis* PC9** | 8.94±0.53 | ***Lpb. pentosus* PC13** | 6.21±0.27 |
| ***Lpb. plantarum* P3** | 10.10±0.03 | ***Lab. brevis* PC3** | 8.76±0.17 | ***Lpb. pentosus* PC4** | 6.15±0.51 |
| ***Lpb. plantarum* NMGL3** | 10.07±0.07 | ***Lab. brevis* PC2** | 8.70±0.15 | ***Lpb. plantarum* NMGL5** | 5.98±0.05 |
| ***Lpb. plantarum* PC10** | 10.06±0.06 | ***Lab. brevis* PC6** | 8.68±0.13 | ***Lab. brevis* JS3** | 5.84±0.31 |
| ***Lpb. plantarum* P2** | 9.88±0.07 | ***Lab. brevis* PC5** | 8.60±0.24 | ***Lab. brevis* JS4** | 5.19±0.52 |
| ***Lpb. plantarum* P5** | 9.82±0.07 | ***Lab. brevis* PC7** | 8.47±0.20 | ***Lpb. plantarum* PC12** | 5.18±0.43 |
| ***Lpb. plantarum* P7** | 9.79±0.20 | ***Lab. brevis* PC8** | 8.45±0.20 | | |
| ***Lpb. plantarum* P1** | 9.71±0.30 | ***Lpb. pentosus* P7** | 7.02±0.20 | | |

Table S2: The gene sequence of *Lpb. plantarum* NMGL2

GGGAGGGCGCGTGCTATAATGCAGTCGAACGAACTCTGGTATTGATTGGTGCTTGCATCATGATTTACATTTG
AGTGAGTGGCGAACTGGTGAGTAACACGTGGGAAACCTGCCCAGAAGCGGGGGATAACACCTGGAAACA
GATGCTAATACCGCATAACAACTTGGACCGCATGGTCCGAGTTTGAAAGATGGCTTCGGCTATCACTTTTGGA
TGGTCCCGCGGCGTATTAGCTAGATGGTGGGGTAATGGCTCACCATGGCAATGATACGTAGCCGACCTGAGA
GGGTAATCGGCCACATTGGGACTGAGACACGGCCCAAACTCCTACGGGAGGCAGCAGTAGGGAATCTTCC
ACAATGGACGAAAGTCTGATGGAGCAACGCCGCGTGAGTGAAGAAGGGTTTCGGCTCGTAAAACTCTGTT
GTTAAAGAAGAACATATCTGAGAGTAACTGTTCAGGTATTGACGGTATTTAACCAGAAAGCCACGGCTAACT
ACGTGCCAGCAGCCGCGGTAATACGTAGGTGGCAAGCGTTGTCCGGATTTATTGGGCGTAAAGCGAGCGCA
GGCGGTTTTTTAAGTCTGATGTGAAAGCCTTCGGCTCAACCGAAGAAGTGCATCGGAAACTGGGAAACTT
GAGTGCAGAAGAGGACAGTGGAACTCCATGTGTAGCGGTGAAATGCGTAGATATATGGAAGAACACCAGTG
GCGAAGGCGGCTGTCTGGTCTGTAACTGACGCTGAGGCTCGAAAGTATGGGTAGCAAACAGGATTAGATAC
CCTGGTAGTCCATACCGTAAACGATGAATGCTAAGTGTTGGAGGGTTTCCGCCCTTCAGTGCTGCAGCTAAC
GCATTAAGCATTCCGCCTGGGGAGTACGGCCGCAAGGCTGAAACTCAAAGGAATTGACGGGGGCCCGCAC
AAGCGGTGGAGCATGTGGTTTAATTCGAAGCTACGCGAAGAACCCTTACCAGTCTTGACATACTATGCAAAT
CTAAGAGATTAGACGTTCCCTTCGGGGACAATGGATACAGTGGTGCATGGTTGTCGTCAGCTCGTGTCGTGA
GATGTTGGATAAGTCCGCAACGAGCGCAACCTTATTATCAGTGCAGCATAAGTGGCACTCTGGTGAGACTGC
CGTGACAACCGAGAGTGGGATGACGTCAATCATCATGCCTATGACTGCTAACACGTGCTACATGATGGTACA
CGAGTTGCGAACCTCGCGAAG

Table S3. The expression levels of low temperature and acid-resistant proteins in *Lpb plantarum* NMGL2 in fermented milk stored at low temperature (4°C) for days 1, 7, 14, and 21.

| | Day 1 Quantity | | | Day 7 Quantity | | | Day 14 Quantity | | | Day 21 Quantity | | |
|---|---|---|---|---|---|---|---|---|---|---|---|---|
| dnaA | 0 | 0 | 0 | 0 | 1218.942 | 1233.82 | 31887.74 | 32372.95 | 34887.93 | 132057.1 | 127528.6 | 132707.8 |
| dnaJ | 3805.299 | 3795.264 | 4291.255 | 4256.374 | 6119.114 | 3727.16 | 92424.61 | 92574.81 | 99434.87 | 359405.6 | 346913.9 | 367442.2 |
| dnaK | 34755.22 | 36828.55 | 33994.82 | 38748.57 | 42968.29 | 40247.76 | 755230.3 | 768227.7 | 828651.6 | 3124662 | 3047459 | 3179973 |
| dnaN | 9623.74 | 7625.477 | 8183.758 | 7353.171 | 7529.269 | 5828.145 | 163949.5 | 162473 | 192376.7 | 703937.8 | 726612.1 | 753248.8 |
| dnaX | 0 | 0 | 0 | 0 | 0 | 0 | 20836.38 | 22556.78 | 23097.51 | 76416.51 | 81724.63 | 81986.83 |
| grpE | 0 | 0 | 0 | 0 | 0 | 0 | 68010.16 | 68599.8 | 72019.2 | 260744.5 | 276313 | 281193.4 |
| groEL | 95403.97 | 99527.36 | 99294.31 | 95845.47 | 97333.36 | 99768.43 | 2308644 | 2240559 | 2507343 | 8320350 | 8115493 | 8557151 |
| rbfA | 3972.366 | 4539.802 | 4723.001 | 1517.154 | 122.2576 | 0 | 70985.29 | 67682.26 | 81461.53 | 289681.4 | 288781.7 | 296821.1 |
| hsp1 | 0 | 0 | 0 | 8906.93 | 7679.882 | 8889.868 | 154467.9 | 147795.5 | 165678.6 | 343556.7 | 346412.6 | 368582 |
| hsp2 | 1762.369 | 1339.543 | 2306.957 | 1915.677 | 1497.806 | 1270.725 | 44610.64 | 43775.53 | 45799.55 | 160636.4 | 176769.6 | 166639.1 |
| hsp3 | 0 | 0 | 4972.727 | 0 | 0 | 0 | 156180.1 | 150562.7 | 169457.5 | 533801.8 | 522632.7 | 559783.6 |
| murA1 | 0 | 0 | 0 | 0 | 0 | 486.2318 | 35503.49 | 31435.05 | 35458.45 | 171943.8 | 180826.2 | 173624.3 |
| murB | 0 | 0 | 0 | 0 | 0 | 0 | 12577.42 | 14110.91 | 15368.56 | 55851.3 | 54802.68 | 56418.31 |
| murC | 0 | 0 | 0 | 0 | 860.3011 | 2584.857 | 33104.51 | 34521.56 | 37088.19 | 102248.6 | 111729.4 | 113298.4 |
| murD | 2000.115 | 2035.173 | 1883.126 | 2404.646 | 2664.79 | 1785.346 | 36012.51 | 38024.61 | 42767.07 | 119724.6 | 127224.4 | 132681.1 |
| murE1 | 1169.105 | 0 | 0 | 0 | 1942.337 | 1676.99 | 63393.44 | 63811.36 | 71858.81 | 232787.8 | 216100.2 | 233090.6 |
| murF | 1471.113 | 1465.111 | 0 | 0 | 0 | 1414.168 | 43052.04 | 44647.62 | 47934.79 | 162139.7 | 164235.7 | 172024 |
| dacA1 | 2900.817 | 3415.7 | 3875.994 | 5337.404 | 5686.645 | 6326.56 | 228692.5 | 224307.6 | 258873.1 | 288471.6 | 297987.6 | 298654.1 |
| dacB | 0 | 0 | 0 | 0 | 0 | 0 | 29276.59 | 27690.86 | 32534.3 | 35962.25 | 34832.12 | 39989.3 |
| cspC | 40200.54 | 36166.39 | 27443.02 | 52015.44 | 52309.02 | 61281.37 | 1696430 | 1822298 | 2056945 | 2847438 | 2729481 | 3183768 |
| gaLE1 | 4372.318 | 4114.339 | 4376.356 | 3086.685 | 3654.22 | 3683.669 | 97415.96 | 97131.82 | 110254.9 | 327683.7 | 333298.2 | 335478.4 |

| | | | | | | | | | | | | |
|---|---|---|---|---|---|---|---|---|---|---|---|---|
| gaLE2 | 13581.7 | 12973.34 | 13678.59 | 8181.834 | 3962.857 | 8698.778 | 301711.7 | 298306.4 | 324952.8 | 1095517 | 1062697 | 1145028 |
| gaLK | 25288.16 | 25348.02 | 24169.04 | 12638.31 | 12480.81 | 11070.14 | 487946.2 | 464839.8 | 531454.4 | 2126685 | 2194526 | 2260797 |
| gaLM1 | 0 | 0 | 0 | 0 | 0 | 0 | 9993.967 | 9949.472 | 15476.97 | 46253.9 | 42983.96 | 47165.31 |
| gaLM3 | 7341.625 | 6477.87 | 7873.891 | 3466.313 | 3954.181 | 4624.281 | 237730.3 | 235703.3 | 264347.5 | 735577 | 699845 | 736947.8 |
| gaLU | 25027.19 | 25950.59 | 25558.96 | 23531.45 | 24250.98 | 24761.41 | 507787.9 | 490675 | 565960.1 | 2073073 | 1907866 | 2013499 |
